# A Freestanding Chitin-Derived Hierarchical Nanocomposite for Developing Electrodes in Future Supercapacitor Industry

**DOI:** 10.3390/polym14010195

**Published:** 2022-01-04

**Authors:** Zheng Dong, Chen Chen, Kaihua Wen, Xiaoyi Zhao, Xihong Guo, Zhongzheng Zhou, Guangcai Chang, Yi Zhang, Yuhui Dong

**Affiliations:** 1 Beijing Synchrotron Radiation Facility, Institute of High Energy Physics, University of Chinese Academy of Sciences, Beijing 100049, China; dongz@ihep.ac.cn (Z.D.); zhaoxiaoyi@ihep.ac.cn (X.Z.); guoxh@ihep.ac.cn (X.G.); zhouzz@ihep.ac.cn (Z.Z.); changgc@ihep.ac.cn (G.C.); 2 School of Nuclear Science and Technology, University of Chinese Academy of Sciences, Beijing 100049, China; 3 State Key Laboratory of High Performance Ceramics and Superfine Microstructure, Shanghai Institute of Ceramics, University of Chinese Academy of Sciences, Shanghai 200050, China; chen.chen@mail.sic.ac.cn; 4 State Key Laboratory of New Ceramics and Fine Processing, School of Materials Science and Engineering, Tsinghua University, Beijing 100084, China; mkh19@mails.tsinghua.edu.cn; 5 Chinese Spallation Neutron Source Science Centre, Dongguan 523808, China

**Keywords:** crustacean cuticle, plywood structure, biochar, conductivity, graphene oxide, chitin

## Abstract

Crustacean cuticles are receiving extensive attention for its potential in developing environmentally friendly and high energy density electrodes for supercapacitor applications. In the current work, the demineralized tergite cuticle of mantis shrimp was employed as a precursor for the fabrication porous biochar. The structural benefits of the cuticle, including the hierarchical nanofiber networks, and the interpenetrating pore systems were maximumly retained, providing a high carbon content and specific surface area scaffold. Graphene oxide sheets were deposited across the biochar through the pore canal systems to further increase the conductivity of the biochar, forming a novel freestanding carbon composite. Throughout the modification process, the material products were examined by a range of methods, which showed desired structural, chemical and functional properties. Our work demonstrates that high performance carbon materials can be manufactured using a simple and green process to realize the great potential in energy storage applications.

## 1. Introduction

Life in today’s world is highly dependent on energy. A vast amount of energy is generated across the world by using traditional fossil fuels. As a direct consequence of the consumption of fossil energy having a devasting impact on the environment and global warming, renewable energy sources, such as solar and wind energy, are increasingly accounting for a significant proportion of the overall energy powering our communities [1,2]. At the same time, the development of powerful energy storage materials for these emerging energy systems, which mainly depend on variable renewable resources, is becoming urgent. Among the many energy storage materials available in the marketplace, batteries are undoubtedly dominating the market due to its superior energy density [3]. However, the fatigue of voltage capacity over time is a key issue, and most of the batteries are generally harmful to the environment and may cause safety risks. Rather than relying on chemical reactions in order to function, capacitors manage to store potential energy electrostatically instead. Capacitors, therefore, present the potential to be an alternative to the storage material community, but they are not yet a substitute for batteries, mainly due to the low energy density. In comparison with the traditional capacitors, such as EDLC and pseudocapacitors, the distances between the two metal plates of supercapacitors are much smaller, resulting in a superior power throughput, and the higher specific surface area in supercapacitors leads to much better power density [4,5,6]. Consequently, supercapacitors are believed to be the most promising alternative to batteries in future urban electric transportation and aerospace industry, where high-speed charging and discharging processes is very important. So far, the supercapacitors are unable to challenge batteries in terms of energy densities; hence, they are mainly applied as complementary equipment to meet the needs of temporary and fast charging [7].

To gain superior efficiency and better sustainability, the supercapacitors need to have high specific surface area and exhibit ultra-strength and light weight [4]. Over the past few decades, substantial efforts have been made to explore new materials for electrodes and electrolytes with properties to retain the benefits of supercapacitors [8,9]. Among them, carbon-based materials (including graphene [10], carbon nanotube [11], etc.) tend to have the better cycle stability and rate capability; therefore, they are considered as the most promising and revolutionary candidates for supercapacitor applications. Due to the high specific surface area, excellent electrical conductivity, and good mechanical and chemical stability, planar open atomic structured graphene materials have drawn extensive attention, and they have found major applications, such as powering major tram systems [12]. The graphene-based materials may need to be stacked into multi-layers or 3D bulk structures for the use as supercapacitors. However, the simply stacked graphene often inevitably suffer significant losses of capacitance and ion permeability due to the hindered ion diffusion between adjacent graphene layers. One of the popular approaches to solve this issue is to agglomerate and deposit single-layer graphene onto a freestanding conductive scaffold material, allowing the ions to freely diffuse across the 3D framework [6,7]. Therefore, searching for porous and mechanically stable scaffolds to maintain the maximum efficiency of graphene becomes the focus of supercapacitor research. Nevertheless, building such a scaffold by chemical synthesis is not an easy task, and the process is usually harmful for the environment. Biomass driven porous carbon materials provide a new path to develop desired scaffolds without severe pollution to the environment [13,14,15,16].

**Figure 1:**
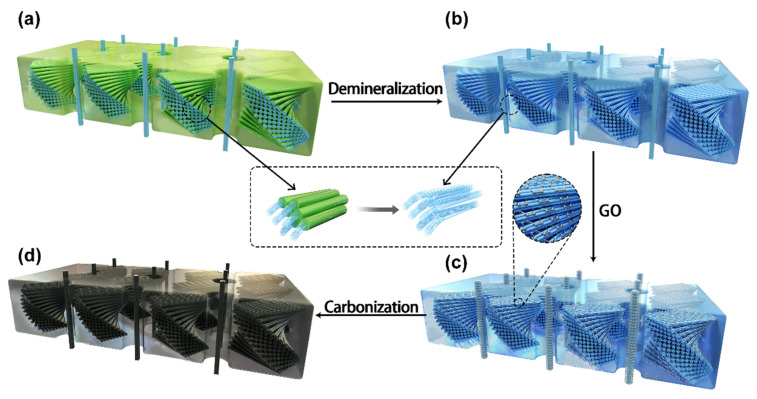
Graphical illustration of the fabrication process of CCF/rGO nanocomposite. (**a**) Schematic illustration of the tergite cuticle showing a combination twisted plywood and interpenetrating pore canal structure formed by mineralized chitin nanofibers; (**b**) Schematic of the residual structure after demineralization, with the inserted picture showing the removal of the encased mineral phases on the chitin nanofiber. (**c**) Schematic showing a 3D demineralized cuticle framework deposited with GO nanoflakes as precursor of CCF/rGO; (**d**) Schematic showing the final self-supporting CCF/rGO obtained after a high temperature heating process.

In the past decade, numerous biological scaffolds, including plants threads [17], animal tissues [15], crustacean cuticles [18], etc., have been employed in the fabrication of novel supercapacitors. The obtained performance varies due to differences on the microstructures and chemical compositions. Generally, the crustacean cuticles are composed by basic materials, such as chitin, protein and minerals, which form a combination of honeycomb-like and interpenetrating twisted plywood-style structure [19,20], as shown in Figure 1a. Compared to the mediocre intrinsic mechanical properties of the raw component materials, the cuticles achieve excellent resistance properties when under powerful and repetitive impacts during preying and ritualized fighting [21]. Various structural and chemical optimization mechanisms have been discovered to favor the toughening and energy dissipation within the crustacean cuticle [22,23,24]. In fact, the hierarchical structure design of crustacean cuticle not only plays an essential role in determining its overall dynamic mechanical function but also can be used as a natural scaffold in manufacturing next generation supercapacitors. Chitin nanofibers, as the main structural building units for the crustacean cuticle, are natural carbon-rich materials and have been applied to prepare heteroatom-doped carbon material for electrode applications. Gao et al. [25], Qu et al. [26], and Huang et al. [27] turned the crustacean cuticle into porous biochar with outstanding capacitance performance, while Qu et al. [28] and Li et al. [29] applied the obtained carbons for the cathodes of batteries. However, these studies turned the cuticle into powders or particles, which abandoned the merits of the self-supporting nature. Based on the crustacean cuticle, Yang et al. [30] and Yu et al. [31] developed novel carbon materials for CO_2_ adsorption and catalyst applications by retaining the porous structure and obtaining high graphitization level through simple chemical modifications. The potential of crustacean cuticle for self-supporting and high efficiency supercapacitors is yet to be explored.

In this study, by using the crustacean cuticle as a 3D scaffold and taking advantage of its interpenetrating and porous structure characteristics, we successfully transformed it into a novel self-supported and highly conductive carbon material through straightforward chemical modification processes (Figure 1). The encased mineral components (principally amorphous calcium carbonate (ACC) and a small amount of amorphous calcium phosphate (ACP) and calcite) in mantis shrimp cuticle were firstly removed by HCl. The products were then immersed in graphene oxide (GO) aqueous solution until the samples were sufficiently encased with GO sheets. After an oven-drying process, the samples were then heated to around 800 °C in a Nitrogen atmosphere, which expelled most of the non-carbon atom within the demineralized cuticle, along with turning the deposited GO into reduced GO (rGO), forming a freestanding composite which retained most of the intrinsic 3D structural properties of the original cuticle and with a high degree of graphitization. The developed material shows excellent performance in conductivity and could easily be manufactured. This would create great potential for developing advanced supercapacitors, strain sensors and battery materials.

## 2. Materials and Methods

### 2.1. Sample Preparation

The tergites of mantis shrimp (*Odontodactylus scyllarus*) were used as precursors for the preparation of nano-porous carbons. The shrimps were obtained from online retailers in Taobao and refrigerated at −80 °C after dissecting. Before demineralization, the cuticle dissected from defrosted specimens were washed thoroughly, cleaned with deionized water to remove impurities, and then dried in a hot air oven at 50 °C for 3 h.

### 2.2. Demineralization Protocols

Demineralization was carried out by immersing the dried cuticle samples in 1 M HCl with agitation speed of 400 rpm for 3 h at room temperature [32,33,34]. The demineralized cuticles were then immersed in deionized water for 30 min, with constant stirring until reaching neutral pH, and dried again in an oven. Finally, the demineralized cuticles were kept in an air-tight container for further use.

### 2.3. Preparation of Graphene Oxide (GO)

GO was prepared following the steps described in the ingeniously modified Hummer’s method [35,36,37]. The expanded graphite (0.5 g) was added to 22 mL of concentrated H_2_SO_4_ with agitation at 0 °C for 5 min. KMnO_4_ (1.5 g) was then slowly added to ensure the temperature of the mixture was kept well below 20 °C. Following this, the obtained solution was further stirred for 5 h at a temperature range of 10 to 15 °C. The mixture was then heated to 30 °C and stirred for another 30 min. After that, 24 mL of deionized water was slowly added into the solution and further stirred for 30 min at 80 °C. The reaction was terminated by adding 4 mL of H_2_O_2_ to remove the excess KMnO_4_. The obtained solution, after adding 30 mL H_2_O, was centrifuged at 4000 rpm for 10 min. Subsequently, the obtained deposition was washed by stirring 200 mL of 5% HCl under a water bath at 40 °C. The deposition was subsequently washed by deionized water until the pH is close to neutral. To facilitate the permeation of GO into the demineralized cuticles, the depositions was ultrasonically crushed in water for 30 min; then, the suspension was centrifuged and lyophilized to obtain GO flakes. The dimension of the GO flakes ranged from 10 to 300 nm determined by atomic force microscopy (AFM). The GO flakes were stored in an air-tight container placed at 4 °C in refrigerator.

### 2.4. Carbonization

The demineralized cuticles were firstly immersed in 5 mg/mL graphene oxide (GO) aqueous solution with gentle shaking at room temperature for 2 days [38,39].The GO impregnated samples were briefly rinsed with distilled water and then dried in an oven at 50 °C for 3 h. Further, the samples were transferred into tube furnace and received high temperature pyrolysis under a constant N_2_ flow, with a heating rate of 5 °C/min up to 800 °C and held for 2 h [31]. After the pyrolysis, the chitin-protein nanofiber (CF) and the GO-deposited chitin-protein nanofiber (CF/GO) turned into carbonized chitin nanofiber (CCF) and carbonized chitin nanofiber with reduced graphene oxide (CCF/rGO), respectively. All the generated CCF and CCF/rGO were taken out and kept in an air-tight container after cooling down to the room temperature.

### 2.5. Material Characterization

The morphological and topographical features of the samples were observed by scanning electron microscopy (SEM, ZEISS Gemini 300, Oberkochen, Germany). This was performed in the secondary electron mode at the acceleration voltage of 3 kV. Nano-CT characterization with volumetric spatial resolution around 64 nm was carried out by using a full-field transmission hard X-ray microscopy system installed at the beamline 4W1A in Beijing Synchrotron Radiation Facility (BSRF, Beijing, China) with a beam energy of 8 keV. Segmentation was conducted with software Avizo. The X-ray diffraction (XRD) measurements were conducted at the beamline 3W1 by using a 2 mm beam (16 keV) at BSRF. An Eigger 1 M detector (DECTRIS AG, Baden-Daettwil, Switzerland) with a pixel size of 75 μm and a resolution of 1030 × 1065 pixels (horizontal and vertical) was used to record the XRD patterns. The natural surface of the cuticle was oriented perpendicular to the X-ray beam. The sample-to-detector distance (about 81.1 mm) was calibrated using silver behenate (AgBe). Small-Angle X-ray Scattering (SAXS) tests were performed at the Time-resolved Ultra Small-Angle X-ray Scattering (USAXS) beamline with a monochromatic beam (beam size: 10 × 10 μm^2^) of 10 keV at the Shanghai Synchrotron Radiation Facility (SSRF, Shanghai, China). The diffraction patterns were collected using a PILATUS 1M detector (DECTRIS AG, Baden-Daettwil, Switzerland) with pixel size of 172 μm and sample-to-detector distance of 3.11 m under exposure time at 1 s. Atomic force microscopy (AFM, Dimension-Edge, Bruker, Berlin, Germany) tests were operated in the tapping mode. Thermogravimetric analysis (TGA, TA TGA55, TA, New Castle, DE, US) was performed in N_2_ atmosphere from 30 to 800 °C with heating rate of 5 °C/min. N_2_ adsorption experiments (ASAP 2460 Version 3.01, Micromeritics, Norcross, GA, US) were conducted at −196 °C after the samples were degassed under vacuum at 200 °C for 6 h. The specific surface area of the samples was measured by using Brunauer-Emmett-Teller (BET) method, and the pore size distribution was calculated using the Barrett-Joyner-Halenda (BJH) method based on adsorption isotherm. X-ray photoelectron spectroscopy (XPS, Thermo Scientific K-Alpha, Louis, MO, US) was carried out using an Al Kα X-ray source with beam size of 400 μm. The electrical conductivity of the materials was measure using a four-point probe system. The lyophilized process of GO was conducted by a vacuum freeze dryer (Biocool, FD-1A-50, Beijing, China) at −50 °C. FTIR tests were conducted on an infrared spectrometer (Thermo Scientific Nicolet iS20, St Louis, MO, US). A total of 16 scans were performed over the range of 4000–550 cm^−1^ at a step size of 4 cm^−1^ under transmittance mode. Raman spectrum were conducted using a Raman spectrometer (Renishaw inVia Raman, Gloucestershire, UK) with a laser wavelength of 473 nm.

## 3. Results and Discussion

### 3.1. D structural Characterization of Mantis Shrimp Cuticle

**Figure 2:**
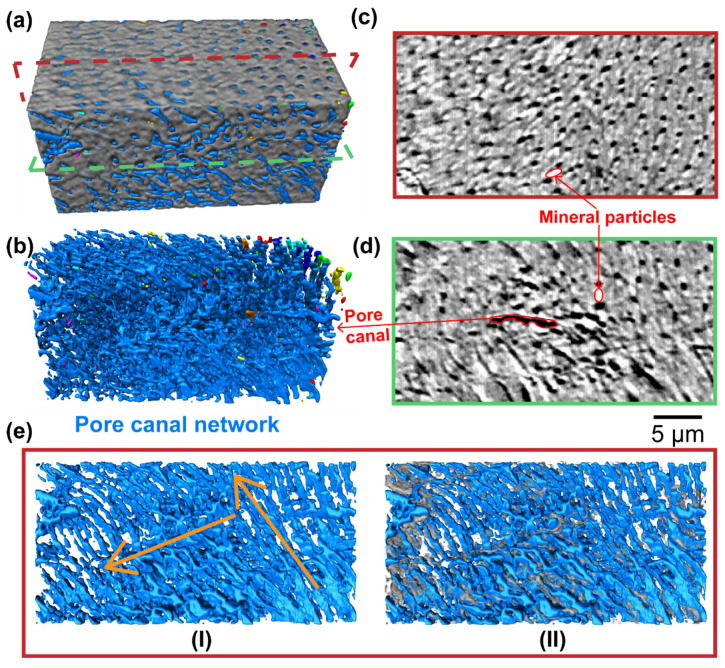
Three-dimensional architecture characterization of the cuticle by nano-CT method. (**a**) A 3D rendering structure of the cuticle showing its three main components: mineral nanoparticle aggregation (white, labeled with red ellipse in (**c**,**d**)), chitin fiber scaffold (grey), and pore canal network (blue). (**b**) The segmented structure of 3D pore canal network; the interpenetrating pore canals were labeled in same color. (**c**,**d**) Slices of reconstructed volume from perpendicular perspective as labeled by red and green dashed box in (**a**), with the pore canals indicated in black as labeled in (**d**). (**e**) A slice of the segmented pore canal structure (I) showing the interconnecting pore canal network and its correlation with the mineral distributions (II); two main orientations of the pore canal system are indicated with orange arrows.

The exoskeleton of crustacean is a multiscale biological material, which is composed by of a chitin-based fibrillar network and reinforced by the incorporation of biomineral particles. These chitin fibrils form a characteristic rotated layered plywood (Bouligand) structure at the scale of ~10 μm, which develops into a well-defined honeycomb lattice-like system with pore canal network running perpendicular to these lamellae [19]. The cuticle of mantis shrimp is a prototype of crustacean exoskeleton with interpenetrating pore canal network (Figure 2a). Nano-CT (3D spatial resolution of 64 nm) is employed to reveal the detailed microstructure of the cuticle for better understanding of the pore canal network. A well-developed 3D pore canal system imaged by nano-CT is presented in Figure 2b, showing a highly connected pore canal system (Figure 2eI). Most of the crustaceans harden the cuticle, not only by sclerotization but also by calcification [40,41], which means they need energetically demanding calcium turnover after ecdysis. The 3D pore canal network serves as channels for material transport during molting, and highly interpenetrating pore canals ensure efficient turnover of calcium. Additionally, as shown in Figure 2c,d, the pore canal contains a long and firm tube with a diameter of about 1 μm, which is reinforced by mineral particles (Figure 2eII). The highly interpenetrating pore canal network makes the cuticle an ideal precursor to prepare hierarchical carbon composites.

### 3.2. Morphology of Composites

**Figure 3:**
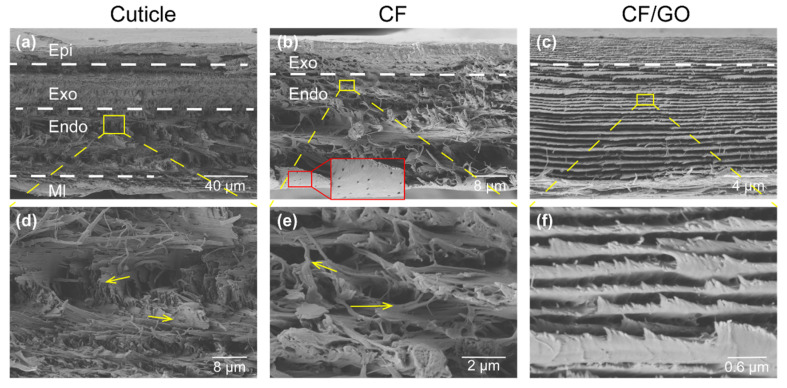
Morphology changes of the cuticle throughout the chemical modification process. (**a**) The SEM image of a cross section of the tergite cuticle, showing its four structurally different layers: epicuticle, exocuticle, endocuticle, and membranous layers. The enlarged image (**d**) showing its dominating twisted plywood structural feature. (**b**) The SEM image collect from demineralized cuticle, showing the retaining of the main structure (enlarged in (**e**)) of the original cuticle, including the pore canals; (**c**) The SEM image showing the structure of demineralized cuticle after immersing in GO aqueous solution and its enlarged image showing in (**f**).

Macroscopically, the pristine cuticle consists of four layers: epicuticle (epi), exocuticle (exo), endocuticle (endo) and membranous (mL) layers (Figure 3a) [19]. Epicuticle is a permeability barrier to the environment which is thin and waxy. As the epicuticle consists mainly of esters of fatty acids and calcite, and no chitin [42], it should be removed after demineralization [34]. Both the exocuticle and endocuticle layers are formed by parallel arrays of mineralized chitin fibers (in-plane fibers, IP), which mainly contribute to the mechanical properties of the procuticle. As shown in Figure 3d, in these two layers, the IP are arranged into a twisted plywood known as Bouligand, which is interpenetrated with the perpendicularly oriented out-of-plane fibers (OP). After demineralization, the chitin molecules were still wrapped by proteins in shells and are responsible for the stability of chitin-protein nanofibers (CF). Eventually, the CF scaffold shows only two layers (Figure 3b): exocuticle and endocuticle. In addition, the twisted plywood (Figure 3e) and honeycomb-like structure (Figure 3b: insert) of mineral chitin fibers with in native cuticle substantially unchanged in those layers after demineralization. After functional with GO, the gaps between the fibers are filled with GO (Figure 3c,f). This excellent coverage is achieved due to strong attractive forces, such as van der Waals and hydrogen bonding between polar oxygen-containing groups of chitin and GO.

**Figure 4:**
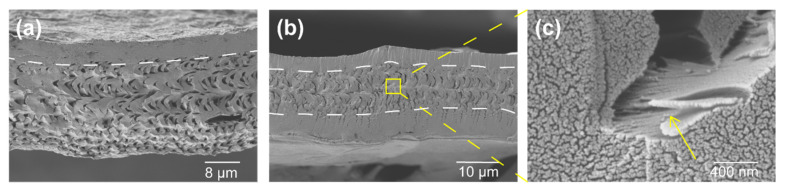
SEM images of the cross-sections showing different morphologies of CCFs and CCF/rGO. (**a**) The morphology of CCF. (**b**) The nanofiber lamellae of CCF/rGO are more tightly packed, and the size of pore canals shrinks. (**c**) The enlarged image showing the CCF/rGO still exhibit layered plywood structure and micro- and nano-sized porous feature.

**Figure 5:**
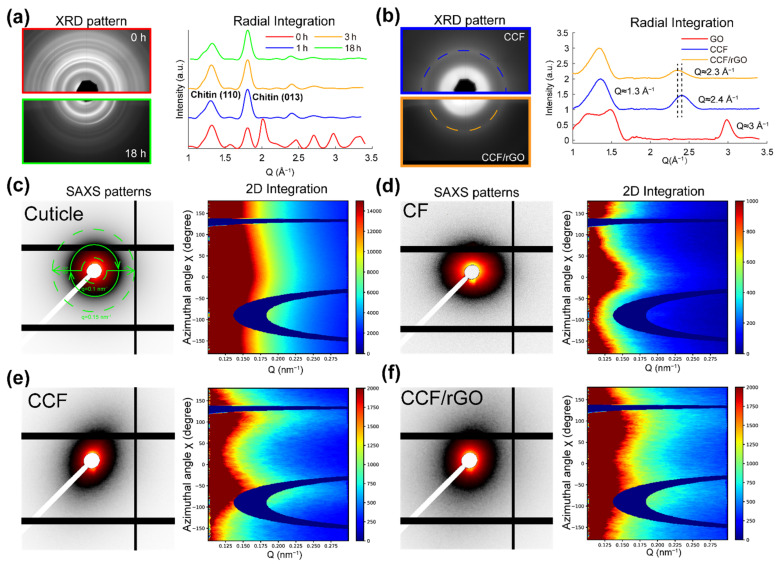
SAXS and XRD examinations of cuticle, CF, CCF, and CCF/rGO. (**a**) Representative XRD patterns from native (top) and demineralized (bottom) tergite cuticle (CF). Integrated intensity of the scattering pattern in the radial direction with scattering vector range (Q) from 1 to 3.4 Å. One-dimensional intensity profiles I(Q) show the typical diffraction peaks changes after the cuticle immersed in 5% HCl for 0 h, 1 h, 2 h and 18 h. The (110) and (013) diffraction of chitin can still be found after demineralization, while the diffraction peaks from mineral phases disappeared after treating with HCl for 1 h. (**b**) Representative XRD patterns of CCF (top) and CCF/rGO (bottom). One-dimensional intensity profiles I(q) acquired from radial integration, showing the typical diffraction peaks from CCF, CCF/rGO and GO. The diffraction peak of CCF at 2.4 Å^−1^ was shifted to 2.3 Å^−1^ in CCF/rGO. (**c**) The representative SAXS patterns of the cuticle and its corresponding 2D azimuthal integration profile, calculating with azimuthal angle range from −180 to 180 degrees and Q range from 0.1 to 0.15 nm^−^^1^. (**d**–**f**): The representative SAXS patterns of CF (**d**), CCF (**e**), CCF/rGO (**f**) and their corresponding 2D azimuthal integration profiles.

CF and GO functional chitin-protein fiber (CF/GO) were used as robust frameworks for the composite synthesis. The pyrolysis temperature has an important influence on the porous structure and graphitic structure of biomass [31]. Increasing the pyrolysis temperature within a certain range will cause the number of pores (especially micropores) and the specific surface area of biomass to increase, which is beneficial to the formation of a good porous structure. At the same time, when the pyrolysis temperature increases, the graphitic structure of biomass will gradually develop, and the whole crystal structure tends to be orderly, resulting in an increase in the degree of graphitization. Yu et al. gained porous graphitic carbon (PSS-bio) by pyrolysis of shrimp shells at different temperatures [31]. They detected that pyrolysis temperature is the key element to regulate the porous structure and carbon configuration of PSS-bio. Based on their conclusion, the pyrolysis condition was set at a heating rate of 5 °C/min up to 800 °C and held for 2 h. With the treatment of high temperature, the precursors simultaneously triggered the transformation of precursor into self-supported electrode materials: carbonization chitin-protein nanofiber (via carbonization) and GO into rGO (via reduction). The ensuing materials are freestanding nano-structured carbon matrixes with 20 μm thickness. Interestingly, porous and the lamellar structure of CF scaffold can be well preserved after high-temperature treatment, and the only difference is that the lamellae spacing became smaller, which can be confirmed by SEM and XRD characterization, as shown in Figure 4 and Figure 5. The micropore-canal networks of CCF and CCF/rGO, respectively, shown in Figure 4a,b, serve as ion channels, which enhance the quick penetration of electrolyte ions into the electrode material [13,16]. The CCF/rGO demonstrate a completely different structure in contrast to CCF. The pore canal network of CCF/rGO was protected with two thin carbon frames, as shown in Figure 5b, which might improve fatigue resistant of carbon material and mechanical stability. With the protection of a thin tablet, stress can be dissipated into the whole pore canal network. Generally, this lamellar structure has a dramatic effect on fracture toughness [43]. The cracks prefer to propagate along the adjacent layers, since propagation across different lamellar layers would need higher stress to tear the fiber sheets.

### 3.3. Structure Characterization

SAXS and XRD are applied to probe the structure changes of the products after demineralization and carbonization. Figure 5a shows the XRD results of cuticles treated with HCl for different hours. As may be seen from the corresponding 1D intensity profile I(Q) curves (Figure 5a), the lattice spacing of chitin D (110) and (013) in demineralized cuticle is not significantly different from the native cuticle, while the peaks from mineral phases vanished after treating with HCl for 1 h. Even after a longer time of HCl treatment (3 h and 18 h), the lattice spacing of chitin still was not significantly different from the native cuticle. The SAXS pattern of native cuticle (Figure 5c) shows a circular scattering pattern, and the scattering intensity mainly came from the ACC and ACP within the cuticle. While the scattering intensity of CF (Figure 4e) mainly came from the plywood structural chitin fibers, both the SAXS and XRD results showed that the structure of chitin fiber remained unchanged during the demineralization process, while amorphous and crystalline mineral phases were removed. The 1D intensity profile I(Q) (Figure 5b) of GO shows a strong diffraction peak at 3 Å^−1^ corresponding to the (004) diffraction of GO, which contributing form the turbostratic structure of GO layers [35]. In addition, the intensity profiles I(Q) of CCF and CCF/rGO both show two distinctive peaks. The peak located at 1.3 Å^−1^ ((002) diffraction) indicates the interlayer structure in the materials. The (100) diffraction of CCF located at 2.4 Å^−1^ corresponds to the triperiodic order of the material [44]. The (100) diffraction of CCF/rGO slightly shifts to 2.3 Å^−1^, which may be caused by the formation of a real graphitic phase. SAXS results of CCF and CCF/rGO are given in Figure 5e,f, respectively. The different colors in the SAXS patterns indicate the variations of the scattering intensities, and the maximum intensity is near the beam stop. Both CCF and CCF/rGO have a scattering pattern that looks similar to ellipse. This indicates the presence of the preferential oriented carbon fiber structure, which means the orientated fiber structure observed in CF was finely maintained after carbonization. The 2D integration profiles (Figure 5e,f) demonstrate that the oriented angle is about 100 degrees for both samples. The retaining of the plywood-style nanofiber arrangement is not only good for its mechanical function but also theoretically helpful in providing conductivity along different directions.

### 3.4. Properties Characterization

**Figure 6:**
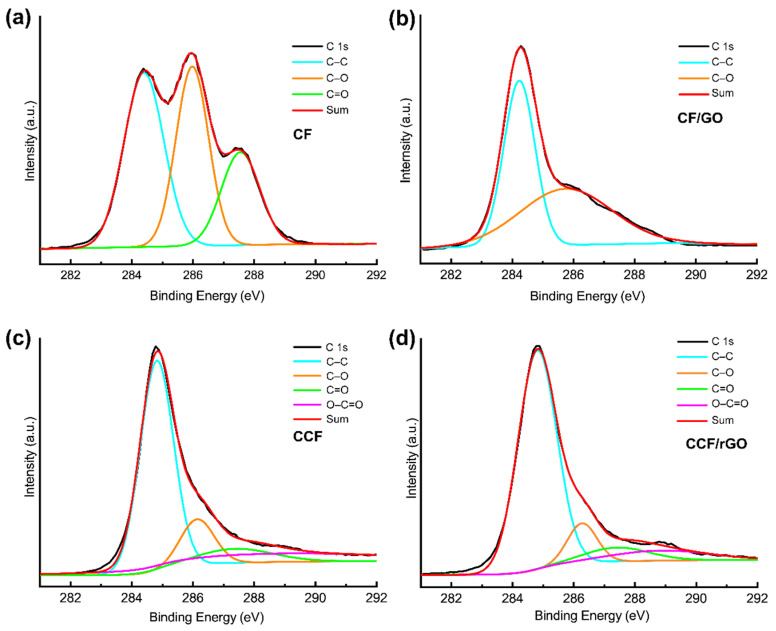
XPS C 1s spectra of CF (**a**), CF/GO (**b**), CCF (**c**), and CCF/rGO (**d**).

XPS analysis provides useful information about the surface elemental composition and functional groups. The C 1s XPS spectra of CCF and CCF/rGO before and after carbonization (Figure 6) were collected to clarify the variations of the electronic state of carbon atom. The C 1s spectrum of CF (Figure 6a) is composed by three sub-peaks at 284.8, 286.1, 287.5 eV, corresponding to the C–C, C–O, and C=O bonds, respectively. The peaks for C–C and C–O bonds show similar intensities, which are stronger than that of C=O bonds. This result complies with the chemical composition of CF. After GO functionalization, the CF/GO shows a typical C 1s spectrum of GO (Figure 6b), indicating that the CF is totally coated by GO. After carbonization, both CCF and CCF/rGO show similar C 1s spectra (Figure 6c,d), mainly containing the C–C peak at 284.8 eV. Some other carbon atoms bearing oxygen-containing groups located at 286.1, 287.5 and 288.8 eV may have resulted from the partial decomposition of the polysaccharide structure [35,45].

**Figure 7:**
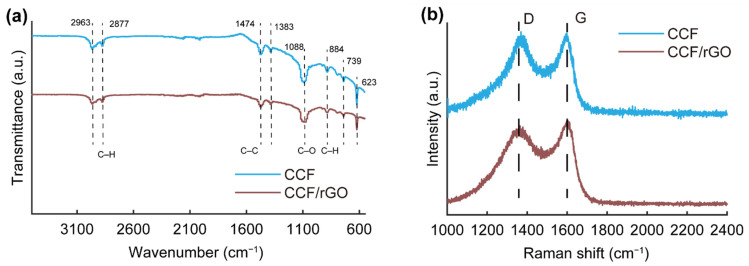
(**a**) FTIR spectra of CCF and CCF/rGO. (**b**) Raman spectra of CCF and CCF/rGO.

The FTIR spectra of CCF and CCF/rGO are shown in Figure 7a. For both samples, the peaks at 2963 and 2877 cm^−1^ are attributed to the stretching vibration of CH_3_, and a peak at 1473 cm^−1^ belongs to the bending vibration of CH_3_. The peaks at 1383 and 739 cm^−1^ are, respectively, attributed to C–H and C–C. However, the peak at 1082 cm^−1^ attributed to the C–O–C skeleton vibration peak from aliphatic alkane structures. The results demonstrate the CCF and CCF/rGO are mainly composed of aliphatic alkane structures [46]. The structural properties of graphitic and non-graphitic carbon were further examined by Raman spectroscopy (Figure 7b). Two characteristic Raman bands for graphite can be found at 1330 (D band) and 1590 (G band) cm^−1^ in the obtained spectra. The D band is related to the structure defection and disorder of carbon, while the G band is corresponding to the crystallinity of graphite [47]. The relative intensity of the D band to the G band (I_D_/I_G_) is usually used as a criterion to determine the degree of disorder for carbon materials. For both samples, the ratio of I_D_/I_G_ were approximately 1, suggesting the formation of graphite structure after higher temperature treatment.

**Figure 8:**
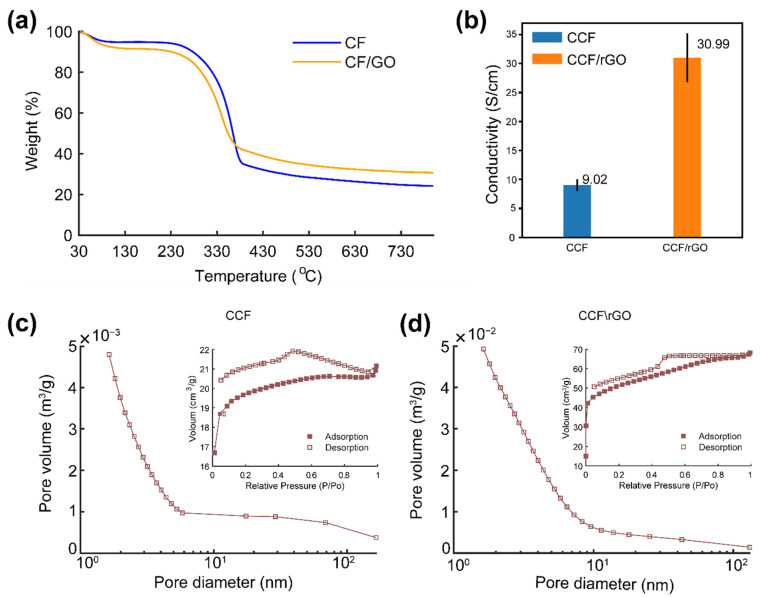
(**a**) TGA of CF and CF/GO. (**b**) Conductivity of CCF and CCF/rGO. (**c**,**d**) Pore size distribution (main image) and nitrogen adsorption/desorption isotherms (inset) of CCF (**c**) and CCF/rGO (**d**).

Further, the TGA curves in Figure 8a reveal the thermal behaviors of CF and CF/GO during carbonization. Both of them show a similar decomposition behavior, demonstrating the major weight loss occurring at the temperature range of 280–400 °C, as shown in Figure 8a, which corresponds to the decomposition of chitin. The depolymerization of chitin occurs simultaneously with dehydration and release of surface functional group. Further, at higher temperature, the transition process of pure sp3-carbon in a tetrahedral amorphous (ta-C) into a completely graphitic (sp2) one (g-C) might occur [31]. The weight yield of CCF/rGO (30.7%) at 800 °C is slightly higher than that of pure CCF (24.2%). This result is due to the flame-retarding behavior of GO in polymeric composites, which prevents the loose release of volatile products from degrading polymer [35].

The specific surface areas and pore-filling behavior of CCF and CCF/rGO were determined by nitrogen-adsorption isotherms. The BET surface area results confirms that both CCF and CCF/rGO composites have a mesoporous structure with a high surface area (Figure 8c,d). The isotherms exhibit an unclosed hysteresis loop with the desorption line under the adsorption line (inserts of Figure 8c,d). This phenomenon was previously observed in some carbonized high-molecular polymers and was ascribed to their complex “throat- or cavity-type” microporous structures [44]. Both isotherms belong to the type IV isotherm, confirming the mesoporous structure of the samples. Compared to the surface area of CCF (~76 m^2^/g), CCF/rGO shows a much higher value of ~187 m^2^/g. The pore-size distribution based on BJH analysis (Figure 8c,d) suggests the pore size of CCF decreases linearly in the range of 1.6–6 nm and centers at approximately 3.3 nm. In contrast, the pore size of CCF/rGO was distribution in the range of 2–42 nm, while the majority of pore size decreases linearly from 1.6–10 nm and centered at approximately 4.2 nm. Micropores (<2 nm) are observed in both samples with high proportion. Hierarchical micro/meso/macro-pores distribution can reduce the diffusion distance for electrolyte ions and decrease the electrical resistance of active biochar materials. Previous studies [13,16,45] have demonstrated that micropores (<2 nm) with ion accessibility can provide large capacitance. In addition, larger pores (2–50 nm) can offer channels for ion transport and reduce diffusion resistance, while macropores (>50 nm) help to form ion buffering reservoirs to decrease the ion transportation distance after electrolyte penetration. The synergistic effects of hierarchically porous structure ensure high affinity of charge transfer and faster ion diffusion process. Hence, the constructions of hierarchical electrodes enable unimpeded ion transportation and good capacitance of supercapacitors.

Carbonization of the CF and CF/GO lead to a strong fusion between the CCF scaffold and graphene sheets in the CCF/rGO composite. The well-distributed rGO sheets act as conductive connectors between CCF. The CCF/rGO composites show an electrical conductivity of 30.99 S/cm (Figure 8b), which is about 3 times higher than that (9.02 S/cm) of the pure CCF. To further evaluate the application potential of the developed composite as supercapacitors, the electrical capacitance of the CCF/rGO composites were characterized using two-electrode cells (described in the Supplementary Note S1). The composites exhibit comparable capacitance properties with previously reported materials [25,26,27].

## 4. Conclusions

In summary, we have developed a novel carbon rich and porous self-supporting nanocomposites for advanced supercapacitor materials from the crustacean cuticle. The guiding principle is to make full use of the properties from its intrinsic interpenetrating nanofiber networks and porous canal system; this will further enhance its conductivity by carbonization and graphitization—all through simple and green material fabrication process. The cuticle was firstly demineralized to get a carbon rich freestanding scaffold composed of chitin-protein nanofibers, followed by utilizing the intrinsic hydrophilic properties of CF and GO, to attach them together and form a composite precursor. In the next stage, the simultaneous abilities of CF and GO to carbonize and to reduce to rGO, respectively, during high temperature treatment are used to obtain the final composite material. In this material, CCFs act as nano spacers in the interpenetrated CCF/rGO network, which enhances the supercapacitor’s excellent volumetric electrochemical performance. A sustainable chitin-derived constituent makes a great ensemble with value-added graphene material. It is possible that further tuning of composite electrode materials based on biomass-derived nanocarbons and valuable additives is the future of cost-effective large-scale production of energy storage devices.

## Data Availability

All raw data that support the findings in this study are available from the corresponding authors upon request.

## Supplementary Materials

The following are available online at https://www.mdpi.com/article/10.3390/polym14010195/s1.
