# Supplementary material for: A Freestanding Chitin-Derived Hierarchical Nanocomposite for Developing Electrodes in Future Supercapacitor Industry
Source: Polymers (Basel). 2022 Jan 4;14(1):195. doi: 10.3390/polym14010195 (PMC8747728; doi:10.3390/polym14010195)
Supplement: Supplementary file 1 [file polymers-14-00195-s001.zip › polymers-1496245-supplementary.pdf]

A freestanding chitin-derived hierarchical nanocomposite for developing electrodes in future supercapacitor industry

Supplementary Information

Zheng Dong [1,2], Chen Chen [3], Kaihua Wen [4], Xiaoyi Zhao [1,5], Xihong Guo [1], Zhongzheng Zhou [1,2], Guangcai Chang [1,2], Yi Zhang [1,2*] and Yuhui Dong [1,2*]

1 Beijing Synchrotron Radiation Facility, Institute of High Energy Physics, University of Chinese Academy of Sciences, Beijing 100049, China
2 University of Chinese Academy of Sciences, Beijing 100049, China
3 State Key Laboratory of High Performance Ceramics and Superfine Microstructure, Shanghai Institute of Ceramics, University of Chinese Academy of Sciences, Shanghai 200050, China
4 State Key Laboratory of New Ceramics and Fine Processing, School of Materials Science and Engineering, Tsinghua University, Beijing 100084, China
5 Chinese Spallation Neutron Source Science Centre, Dongguan 523808, China
* Correspondence: yizhang88@ihep.ac.cn (Y.Z.); dongyh@ihep.ac.cn (Y.D.)

Supplementary Figures

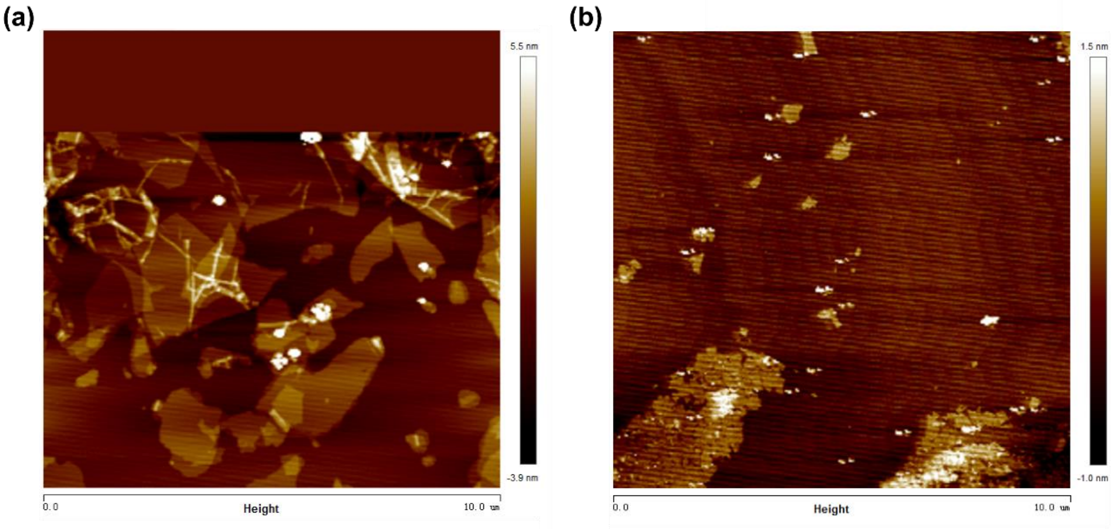

Supplementary Figure S1. Atomic force microscopy (AFM) figures of graphene oxide (GO) flakes before (**a**) and after (**b**) ultrasonically crushed in water for 30 min.

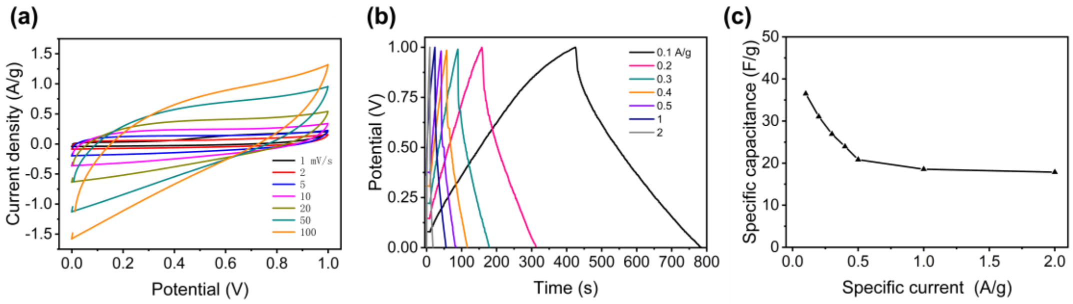

Supplementary Figure S2. Electrochemical characterizations of CCF/rGO in 6 M KOH solution at atmosphere temperature. (**a**): Cyclic voltammograms (CV) at various scan rates from 5 to 100 mV s^{-1}. (**b**): Charge-discharge cures with current density from 0.1 to 2 A/g. (**c**): Specific capacitances at different current densities.

Supplementary Note

Supplementary Note S1: Electrochemical Measurements of CCF/rGO

The electrochemical properties of as-obtained samples were investigated using a two-electrode cell at room temperature. In order to assemble electrodes suitable for electrochemical measurements, the CCF/rGO was crushed using a grinder and dissolved in HNO_3 (1 M) with stirring for 6 h. And then the sample was washed multiple times by deionized water until neutral PH. After dried in an oven at 60 °C, the prepared powders were mixed with polytetrafluoroethylene (PTFE), and acetylene black according to the mass ratio of 8:1:1 for preparing of working electrodes. A small amount of ethanol was added to the mixture to produce a homogeneous paste. The mixture was pressed onto nickel foam current collectors (1.5 cm in diameter) to make electrodes. Before the electrochemical test, the as-prepared electrode was soaked overnight in a 6 M KOH electrolyte. Two symmetrical work electrodes were assembled and tested at different cell voltages. The cyclic voltammetry (CV) measurement was conducted on a CHI660E (Shanghai, China), and the galvanostatic charge-discharge measurement was performed on a Land CT2001A cycler at room temperature (Wuhan Land Instrument Company, China).

The electrochemical measurements of the resultant carbons were performed in 6 M KOH electrolyte using two-electrode cells (Fig. S2). Fig. S2-a shows the cyclic voltammetry (CV) curves of the obtained carbons at the voltage range of 0–1 V at different scan rates from 1 to 200 mV s^{-1}. The CV curves demonstrate scan rate dependence for the composite electrode. The quasi-rectangular loop curves at various scan rates from 5 to 100 mV s^{-1} reveal the quick electrochemical response and domination of double-layer capacitance which accounts for the main part. The galvanostatic charge/ discharge (GCD) curves of the resultant carbons with current density from 0.1 to 2 A/g are shown in Fig. S2-b. It can be found that all curves formed a roughly equicrural triangular shape, indicating the superior reversibility of prepared electrodes during the working condition, whereas the existence of some distortions on the curves was due to the presence of pseudocapacitance. In addition, the rate performance indicates the duration of charge, which is in agreement with the CV results. The specific capacitances at various current densities were shown in Fig. S2-c, which represents the rate capability of resultant carbons. The achieved capacitances were 37, 31, 27, 24 ,21, 19 and 18 F g^{-1} under current density of 0.1, 0.2, 0.3, 0.4, 0.5, 1.0 and 2.0 A g^{-1}, respectively. The electrochemical measurements demonstrate that developed CCF/rGO composite have great potential for high efficiency supercapacitors application.
